# Age‐dependent variation of female preponderance across different phenotypes of multiple sclerosis: A retrospective cross‐sectional study

**DOI:** 10.1111/cns.13083

**Published:** 2018-11-08

**Authors:** Andrei Miclea, Anke Salmen, Greta Zoehner, Lara Diem, Christian P. Kamm, Panos Chaloulos‐Iakovidis, Marius Miclea, Myriam Briner, Kostas Kilidireas, Leonidas Stefanis, Andrew Chan, Maria Eleftheria Evangelopoulos, Robert Hoepner

**Affiliations:** ^1^ Department of Neurology Bern University Hospital and University of Bern Bern Switzerland; ^2^ Department of Neurology, Neurology and Neurorehabilitation Center Luzerner Kantonsspital Lucerne Switzerland; ^3^ Neurological Outpatient Department Neurocenter Peine Peine Germany; ^4^ Department of Neurology, Eginition University Hospital National and Kapodistrian University of Athens Athens Greece; ^5^ Department of Neurology, St. Josef Hospital Ruhr University Bochum Bochum Germany

**Keywords:** aging, Multiple sclerosis, sex distribution, sex hormones

## Abstract

**Introduction:**

Multiple sclerosis (MS) is an autoimmune disease of the CNS, which predominantly affects women. Studies investigating the sex distribution in MS are sparse. We aim to analyze the female‐to‐male ratio (F/M ratio) in different MS phenotypes in association with age at diagnosis and year of birth.

**Methods:**

We performed a retrospective cross‐sectional analysis by cumulating data (sex, year of birth, age at diagnosis, and MS phenotypes) from unpublished and published studies of the participating centers.

**Results:**

Datasets of 945 patients were collected. The overall F/M ratio was 1.9:1.0 and female preponderance was present in all phenotypes except for primary progressive MS (PPMS), in which men were predominantly affected (F/M ratio: 0.5:1.0). Female preponderance declined with increasing age at diagnosis and was no longer present in relapsing‐remitting MS (RRMS) patients > 58 years of age.

**Conclusion:**

Our data demonstrate an age dependency of female preponderance in MS except for PPMS. This could be influenced by the lifecycle of sex hormone secretion in women. In PPMS, a male preponderance was observed in all age‐groups, which might point to pathophysiological mechanisms being less influenced by sex hormones.

## 1 INTRODUCTION

Similar to other autoimmune diseases, multiple sclerosis (MS) has a female preponderance.^1^ The prevalence of MS and the female‐to‐male ratio (F/M ratio) in MS have increased over the last decades, while age at MS onset has decreased.^2^ Several etiological factors associated with modern lifestyle are considered as possible reasons for shifts in MS prevalence, F/M ratio, and age at onset.^2^, ^3^ Whereas the relapsing‐remitting (RR) disease course has a female preponderance, the primary progressive MS (PPMS) phenotype has a slight male preponderance with approximately 60% of patients being male.^5^ Summarizing data from different studies, it appears that age at onset influences sex distribution of RRMS patients (F/M ratio at MS onset: <10 years (y) 1.4:1.0, 18‐49 y 3.1:1.0 and 50‐59 y 2.3:1.0).^6^, ^7^ Nevertheless, studies comparing the sex distribution between different MS phenotypes and investigating the influence of age at diagnosis on the sex ratio are sparse.

The aim of the present study was (a) to assess female preponderance in a European cross‐sectional patient cohort, (b) to examine changes in the F/M ratio throughout the last decades, and (c) to evaluate sex ratios in different MS phenotypes. This may provide a better insight into the impact of sex hormones on pathophysiological mechanisms involved in relapsing and progressive disease courses.

## 2 METHODS

We collected data of patients treated at European MS centers located in Athens (Greece), Bern (Switzerland), Bochum (Germany), and Peine (Germany). In the participating centers, data were gathered from medical records and from previously published retrospective MS studies with different nonepidemiological scopes.^8^, ^9^ The study was approved by the responsible local ethics committees (Eginition Hospital, Athens University Medical School; Bern University Hospital: KEK‐BE 2017‐01369; Ruhr‐University Bochum: 5408‐15; Medical Association of Lower Saxony (06/17/16)). The following data were assessed: year of birth, age at diagnosis, sex, and MS phenotype. MS diagnoses were included as given in the records, that is, in accordance with the MS diagnostic criteria used at the respective time of diagnosis.

Age was used as a continuous variable presented as mean and standard deviation (SD), whereas year of birth was categorized for our analysis. Continuous variables were analyzed using the Mann‐Whitney test (MWT) or Kruskal‐Wallis test for comparisons of more than two groups. A *P*‐value <0.05 was defined as significant.

For the estimation of the effect of age at diagnosis on the sex ratio, we performed a logistic regression analysis with sex (dichotom: 0 = male, 1 = female) as dependent and age at diagnosis as independent variable (continuous), which was run separately for the four different disease courses (radiologically isolated syndrome (RIS), clinically isolated syndrome (CIS), RRMS, and PPMS). In the regression analysis, we defined significance as a *P*‐value <0.013 following Bonferroni's adjustment for multiple testing. Finally, the predicted probabilities of female sex in all four disease courses were plotted against age at diagnosis to identify an age cut off for the switch of sex preponderance from female to male*.*

## 3 RESULTS

**Figure 1:**
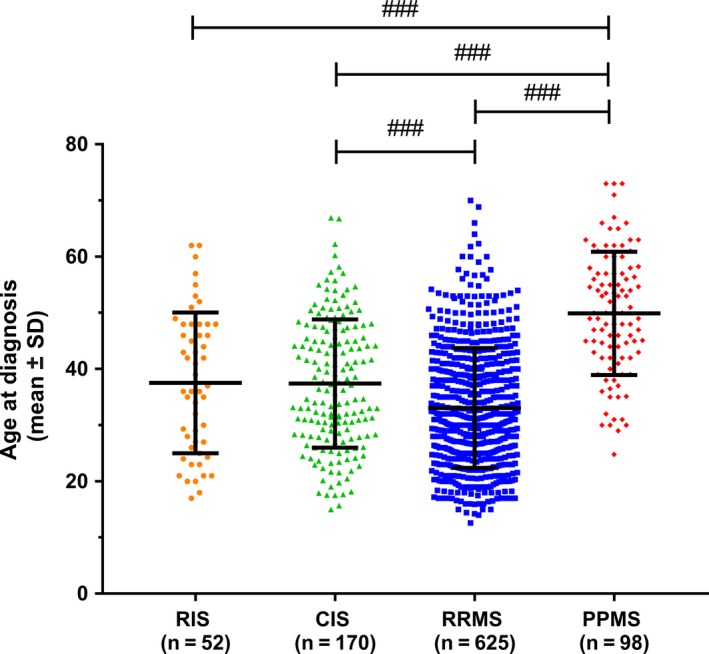
Age at disease diagnosis (mean ± standard deviation (SD)). Statistics: Kruskal‐Wallis test. ^###^ *P* < 0.001.

In total, 945 patients were retrospectively analyzed (see Figure S1 for the distribution across centers). Age distribution was significantly different between MS phenotypes. Compared to all other MS phenotypes, PPMS patients were older at diagnosis (Figure 1).

**Figure 2:**
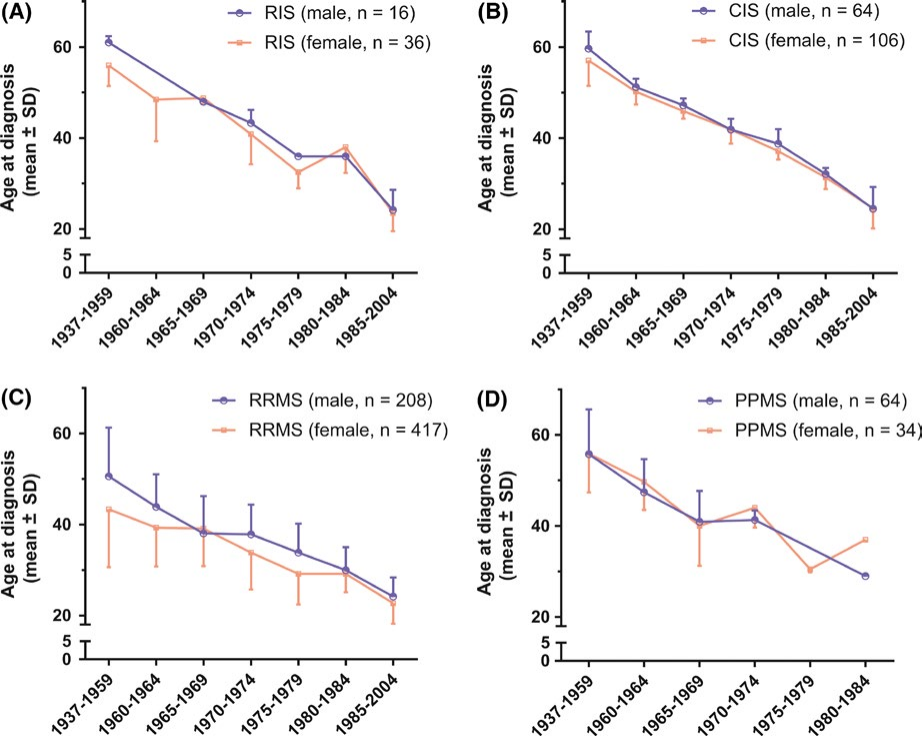
Age at diagnosis (mean ± standard deviation (SD)) in regard to year of birth of female and male (A) RIS, (B) CIS, (C) RRMS, and (D) PPMS patients. Each symbol represents a data point for the given episode. If a symbol is not given, data are missing for this time interval

**Figure 3:**
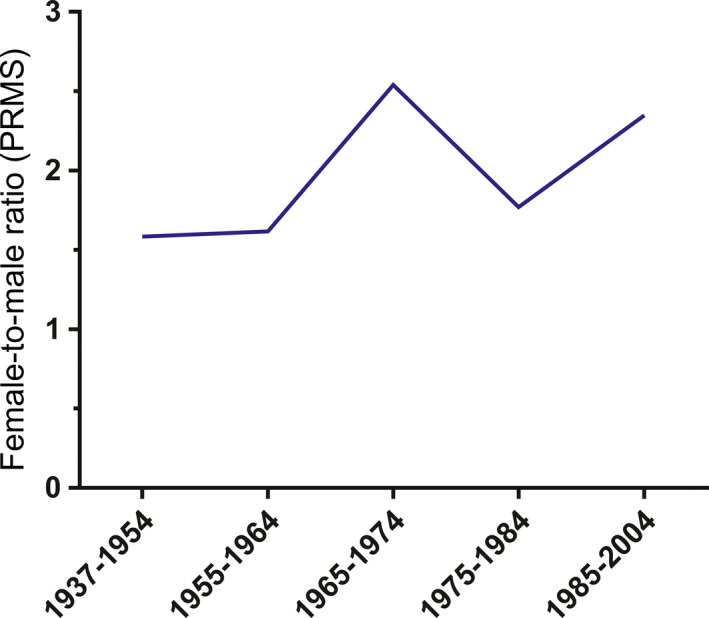
Female‐to‐male ratio in RRMS patients stratified by year of birth. Note: Female‐to‐male ratios not given for other MS phenotypes due to low patient numbers

**Figure 4:**
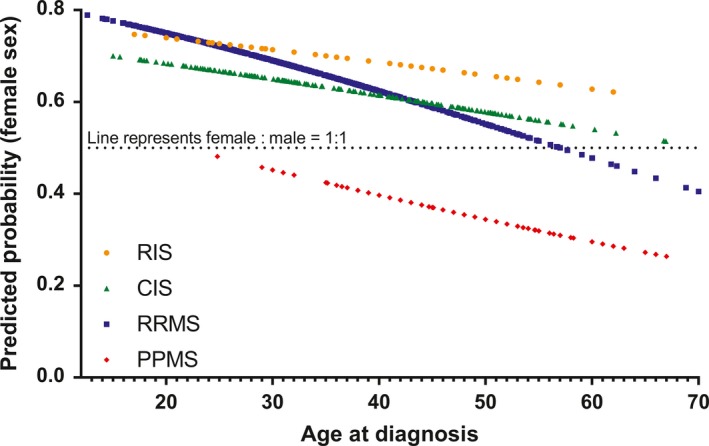
Predicted probability of female sex in regard to age at diagnosis. Predicted probabilities were obtained by respective logistic regression analysis, which is presented in detail in Table 2

Considering all MS phenotypes, female patients (mean 34.0, SD 11.4) were younger than male patients at diagnosis (mean 38.9, SD 12.6; MWT *P* < 0.001). In all disease courses analyzed, age at diagnosis decreased in both genders with a more recent year of birth (Figure 2). The overall F/M ratio irrespective of MS phenotype was 1.9:1.0. It was highest in RIS (2.3:1.0) and RRMS (2.0:1.0) and lowest in PPMS patients (0.5:1.0; Table 1). Stratifying RRMS patients by the year of birth, an increase in the F/M ratio with a more recent year of birth was noted (Figure 3). Using logistic regression analysis, we demonstrated that age at RRMS diagnosis is significantly associated with sex distribution with a lower likelihood of female sex with older age at diagnosis (Table 2). In RRMS patients, this creates a cut off at 58 years of age at diagnosis after which the F/M ratio turns from a female to a male preponderance which is not found in other disease courses (Figure 4).

**Table 1:** Gender distribution for different MS phenotypes and for the age cut off of the RRMS group

Disease course	Female (%)	Male (%)	Total	Female‐to‐male ratio
RIS	36 (69%)	16 (31%)	52	2.3:1.0
CIS	106 (62%)	64 (38%)	170	1.7:1.0
RRMS	417 (67%)	208 (33%)	625	2.0:1.0
PPMS	34 (35%)	64 (65%)	98	0.5:1.0
Total	593 (63%)	352 (37%)	945	1.9:1.0
RRMS				
≤58 years	412 (67%)	203 (33%)	615	2.0:1.0
>58 years	5 (50%)	5 (50%)	10	1.0:1.0

**Table 2:** Logistic regression analysis. Gender (dichotom male = 0/female = 1) is the dependent variable and age at diagnosis (continuous) the independent variable. Statistic: Logistic regression analysis

	OR	95% CI	*P*‐value	*R* ^2^
RIS	0.99	0.94‐1.04	0.59	0.01
CIS	0.99	0.96‐1.01	0.28	0.01
RRMS	0.97	0.96‐0.99	**<0.001**	0.02
PPMS	0.98	0.94‐1.02	0.25	0.01

Following Bonferroni's adjustment, a significance can be assumed if *P*‐value <0.013. Significant *P*‐values are marked in bold.

95% CI, 95% confidence interval; OR, odds ratio.

## 4 DISCUSSION

The results of this study support previous findings on the increase in female preponderance over the last decades.^2^, ^12^, ^13^ This might reflect rather a general epidemiological phenomenon than a disease‐specific trait. Moreover, we demonstrate that female preponderance depends on younger age at diagnosis. Similarly, a female preponderance (2.5:1.0) in MS patients between 20 and 40 years of age but a male preponderance (1.0:1.5) in patients >50 years was previously reported.^14^ A decreasing age at diagnosis, which might contribute to an increase in female preponderance, was also reported for the Norwegian county of Buskerud.^15^ The decreasing age at diagnosis may partially be explained by changes in MS diagnostic criteria and broader availability of diagnostics. In line with previous studies.^15^, ^16^ an increasing female preponderance was also reported by a more recent epidemiological study from Denmark: female incidence did not only increase generally in MS, but more pronounced in patients with late disease onset.^18^ However, differences in female preponderance between CIS, RRMS, and PPMS had not been investigated. As demonstrated in our patient cohort, PPMS patients were older and had a slight male preponderance, which is also supported by other cohort studies.^12^, ^19^ The female preponderance in RRMS may be owed to sex differences in immune function driven by hormonal factors, which predispose to stronger autoimmune responses in women.^20^ On the contrary, the neurodegenerative component and the progression of disability accumulation seem less pronounced in female compared to male patients, which may explain higher susceptibility of male patients for progressive disease courses like PPMS.^21^, ^22^ This is further reinforced by experimental evidence: in experimental autoimmune encephalomyelitis, male mice have a more severe Wallerian degeneration in the central nervous system than females.^23^ Another possible explanation is that with increasing age, men exhibit a higher decrease in the expression of genes in brain regions associated with anabolic pathways, including mitochondrial energy production and protein synthesis than age‐matched women.^24^ These pathways can be linked to mitochondrial dysfunction, which is an important factor in MS‐associated neurodegeneration.^25^

In the following, limitations of our study will be discussed. As the RRMS subgroup represents approximately 66% of all included patients (n = 945), the other three subgroups and here especially the numbers of included male patients were relatively small. Thus, a convincing calculation of the F/M ratio by categorized year of birth was only possible for RRMS patients. Further, because nearly 90% of the patients were from German or Swiss MS centers, a sampling bias with a predominant inclusion of MS patients from these countries has to be taken into account. Another factor limiting data interpretation is the heterogeneity of MS diagnostic criteria used over time in patients included in this study, since these were subject to substantial changes over the last decades.

To conclude, our data demonstrate a varying female preponderance in different MS disease groups. Furthermore, except for the PPMS group, an age dependency of female preponderance was shown. Differences between MS phenotypes may provide further insights into pathophysiology with female sex hormones being associated with stronger autoimmune responses while male sex hormones may predispose to neurodegeneration during the progressive phase of the disease.

## CONFLICT OF INTEREST

A Miclea reports no disclosures. A Salmen received speaker honoraria and/or travel compensation for activities with Almirall Hermal GmbH, Biogen, Merck, Novartis, Roche and Sanofi Genzyme, none related to this work. P Chaloulos‐Iakovidis, G Zoehner report no disclosures. L Diem received travel grants from Merck, Biogen, Roche, and Bayer Schweiz. CP Kamm has received honoraria for lectures as well as research support from Biogen, Novartis, Almirall, Bayer Schweiz AG, Teva, Merck, Sanofi Genzyme, Roche, Celgene, and the Swiss MS Society (SMSG). M Miclea received travel grants from Novartis, Biogen Idec, Bayer, Teva, and Merck Serono. K. Kilidireas has received travel grants and consulting fees from Biogen, Novartis, Genzyme, Teva, and Merck. L Stefanis reports no disclosures in relation to this work. M Briner received travel grants from Merck. A Chan has received personal compensation for activities with Bayer, Biogen, Genzyme, Merck, Novartis, Roche, Teva. He received research support from the Swiss National Fonds (SNF, No. 310030_172952), Genzyme, and UCB. ME Evangelopoulos has received travel grants and consulting fees from Biogen, Novartis, Genzyme, Teva, and Merk. R Hoepner received research and travel grants from Novartis and Biogen Idec. He also received speaker's honoraria from Biogen, Novartis, Merk, and Almirall.

## Data Availability

The authors are willing to share the data of these populations for collaborations. Please contact the corresponding author.
