# Supplementary figures and images for: Age‐dependent variation of female preponderance across different phenotypes of multiple sclerosis: A retrospective cross‐sectional study
Source: CNS Neurosci Ther. 2018 Nov 8;25(4):527–31. doi: 10.1111/cns.13083 (PMC6488902; doi:10.1111/cns.13083)

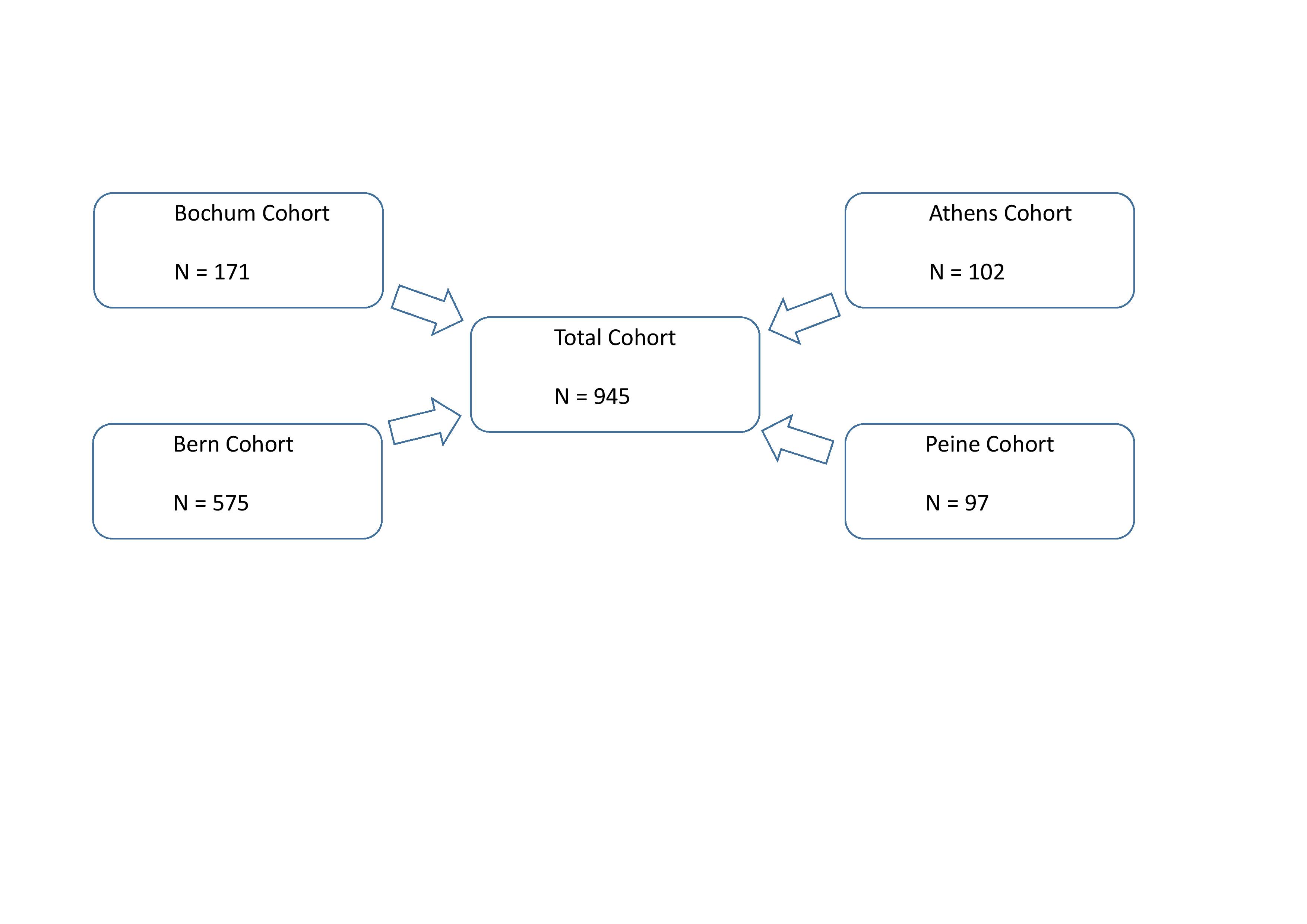

Supplement: Supplementary file 1 [file CNS-25-527-s001.jpg]
